# To develop a regional ICU mortality prediction model during the first 24 h of ICU admission utilizing MODS and NEMS with six other independent variables from the Critical Care Information System (CCIS) Ontario, Canada

**DOI:** 10.1186/s40560-016-0143-6

**Published:** 2016-02-29

**Authors:** Raymond Kao, Fran Priestap, Allan Donner

**Affiliations:** Department of National Defense, Royal Canadian Medical Services, 1745 Alta Vista Drive, Ottawa, K1A 0K6 Ontario Canada; London Health Sciences Center, Divisions of Critical Care and Robarts Research Institute, Western University, 800 Commissioner’s Rd E., London, Ontario N6A 5W9 Canada; Harvard School of Public Health, Harvard University, 677 Huntington Ave., Boston, 02115 MA USA

**Keywords:** Intensive care unit (ICU), Mortality, MODS, NEMS, Logistic regression, Hosmer and Lemeshow goodness-of-fit test

## Abstract

**Background:**

Intensive care unit (ICU) scoring systems or prediction models evolved to meet the desire of clinical and administrative leaders to assess the quality of care provided by their ICUs. The Critical Care Information System (CCIS) is province-wide data information for all Ontario, Canada level 3 and level 2 ICUs collected for this purpose. With the dataset, we developed a multivariable logistic regression ICU mortality prediction model during the first 24 h of ICU admission utilizing the explanatory variables including the two validated scores, Multiple Organs Dysfunctional Score (MODS) and Nine Equivalents Nursing Manpower Use Score (NEMS) followed by the variables age, sex, readmission to the ICU during the same hospital stay, admission diagnosis, source of admission, and the modified Charlson Co-morbidity Index (CCI) collected through the hospital health records.

**Methods:**

This study is a single-center retrospective cohort review of 8822 records from the Critical Care Trauma Centre (CCTC) and Medical-Surgical Intensive Care Unit (MSICU) of London Health Sciences Centre (LHSC), Ontario, Canada between 1 Jan 2009 to 30 Nov 2012. Multivariable logistic regression on training dataset (*n* = 4321) was used to develop the model and validate by bootstrapping method on the testing dataset (*n* = 4501). Discrimination, calibration, and overall model performance were also assessed.

**Results:**

The predictors significantly associated with ICU mortality included: age (*p* < 0.001), source of admission (*p* < 0.0001), ICU admitting diagnosis (*p* < 0.0001), MODS (*p* < 0.0001), and NEMS (*p* < 0.0001). The variables sex and modified CCI were not significantly associated with ICU mortality. The training dataset for the developed model has good discriminating ability between patients with high risk and those with low risk of mortality (c-statistic 0.787). The Hosmer and Lemeshow goodness-of-fit test has a strong correlation between the observed and expected ICU mortality (*χ*^2^ = 5.48; *p* > 0.31). The overall optimism of the estimation between the training and testing data set ΔAUC = 0.003, indicating a stable prediction model.

**Conclusions:**

This study demonstrates that CCIS data available after the first 24 h of ICU admission at LHSC can be used to create a robust mortality prediction model with acceptable fit statistic and internal validity for valid benchmarking and monitoring ICU performance.

**Electronic supplementary material:**

The online version of this article (doi:10.1186/s40560-016-0143-6) contains supplementary material, which is available to authorized users.

## Background

Patients in the intensive care units (ICUs) have a heterogeneous disease processes and illness severity. Scoring systems developed for ICU patients were introduced 34 years ago with the goal of using physiologic data available at ICU admission to predict individual patient outcomes. Although these predictions have little utility for managing individual patients, they do provide a mechanism for assessing ICU performance by comparing the actual outcome in a given population to the expected outcome determined by the prediction algorithms. The scores that assess the disease severity on admission and are used to predict outcome include the Acute Physiology and Chronic Health Evaluation (APACHE) [1–3], the Simplified Acute Physiological Score (SAPS) [4], and the Mortality Prediction Model (MPM) [5]. The organ dysfunction scores that assess the presence and severity of organ dysfunction include the Multiple Organ Dysfunction Score (MODS) [6] and Sequential Organ Failure Assessment (SOFA) [7]. The score that assesses nursing workload is the Therapeutic Intervention Scoring System (TISS) [8], and the Nine Equivalents of Nursing Manpower Use Score (NEMS) [9] (Additional file 1: Table S1) assesses ICU resource utilization and efficiency. Many of these measurement systems involve resource-intensive data collection.

In 2007, the Critical Care Services Ontario (CCSO), a division of the Ontario Ministry of Health and Long Term Care, developed the Critical Care Information System (CCIS). The purpose of CCIS is to provide the Ministry, Local Health Integration Networks (LHINs) and hospitals with information on bed availability, critical care utilization, and patient outcomes. The CCIS uses a web-based application to collect real-time information on every patient admitted to a critical care unit in Ontario acute care hospitals. Data captured includes, but it is not limited to the following: demographics, admission and discharge details, MODS on admission, daily NEMS, and patient outcomes such as ICU mortality and other outcomes associated with quality of care.

The MODS is an objective scale that quantifies the severity of multiple organ dysfunction for patients admitted to critical care. The score reflects six major organ systems and the specific physiological data associated with each system [6]. A total of 0–4 points are assigned to each system, where a score of 0 is normal and 4 is the most dysfunctional to give a total maximum score of 24. MODS was not designed to predict mortality, but an increasing MODS does correlate with ICU outcome [6].

The NEMS was developed from the TISS-28 score and is a less complicated and is more widely used to measure resource utilization in critical care [10]. The score is determined based on the need for any of the nine life support interventions. A weighted point is awarded to each of the nine categories to give a maximum score of 56. NEMS has been validated in large cohorts of ICU patients and is easy to use with minimum inter-observer variability [11]. It has been utilized to classify the different levels of ICUs based on nursing workload efficacy as distinguished from the amount of care being provided.

In an effort to help hospitals analyze and interpret their data, CCSO produces and distributes quarterly reports that include a multitude of utilization and quality indicators of which one is ICU mortality. This data is presented in a manner that promotes benchmarking, but there is currently no means of risk adjustment to ensure that units are comparing themselves to centers with similar case mix and illness severity. Review of these reports shows that there are units with direct correlation of higher mean MODS and ICU mortality, but this is not always the case. There are units with similar MODS but differing mortality rates. The objective of this study is to investigate if existing CCIS data collected by the Critical Care Trauma Center (CCTC) and Medical-Surgical Intensive Care Unit (MSICU) of London Health Sciences Centre (LHSC) can be used to develop and validate an acceptable ICU mortality prediction model that might improve current performance measurement reporting.

## Methods

### Study design and patient population

This is a retrospective study of two adult intensive care units at the LHSC, an academic teaching facility, affiliated with The University of Western Ontario. The CCTC is a 30-bed general medical, surgical, trauma, and oncological unit, and the MSICU is a 25-bed unit that specializes in the care of various patient populations including neurosurgical, cardiovascular surgery, and transplantation patients. In both units, the care is provided by multidisciplinary teams of professional health care providers and is directed by physicians that have specialty training in critical care. This study was approved by the Western Health Research Ethic Board on 13 Nov 2013, IRB 00000940.

Between 1 Jan 2009 to 30 Nov 2012, data was prospectively collected on 4784 admissions to the CCTC and 4297 admissions to the MSICU. The primary endpoint of this study is to develop a mortality prediction model utilizing the available data from CCIS during the first 24-h ICU admission.

### Data sources

Relevant data from both units was exported from CCIS. Comorbidities are not included in CCIS but it is important because it may delay diagnosis, influence treatment decision, are related to complications, may influence chances of survival, and can confound analysis [12]. Based on the APACHE II mortality prediction model, comorbidities can reflect diminished physiological reserve; thus, it is important to incorporate past relevant medical/surgical history into a mortality prediction model. All Canadian hospitals submit information to the Canadian Institute of Health Information (CIHI) which is an independent, not-for-profit corporation that aims to contribute to the improvement of the health of Canadians and the health care system by disseminating quality health information. The CIHI utilizes the Charlson Comorbidity Index (CCI) as their measure of comorbidity [13] which has been shown to be highly associated with 1-year patient mortality and has been widely used in clinical research [14]. To obtain comorbidities for the prediction model, the ICD-10-CA data for patients admitted to either of the critical care units during the time frame of interest was obtained from the LHSC Health Records Department and only type 1 diagnoses, which specifically refer to pre-admission comorbidity was utilized.

### Data management

**Fig. 1:**
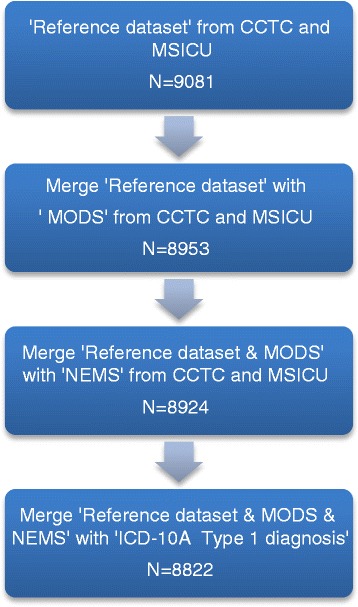
Data management flow chart. Reference admit/discharge dataset, *N* = 9081 records. Merged reference dataset with MODS and NEMS dataset resulted in *N* = 8924 records. Merged CCIS dataset (*N* = 8924) with type 1 diagnosis resulted final dataset for analysis, *N* = 8822 records. Total 2.9 % records were not matched

The data from CCIS was exported in three parts. The reference dataset (*N* = 9081) contains demographic, admit/discharge date and time, admitting diagnosis, and the source of admission of the patients. The second part of the data is the MODS score on the day of admission to the ICU, and the third part of the data is the NEMS score on the day of admission to the ICU. The medical record number (MRN) and ICU admission date were used as the common linking variables to merge the MODS and NEMS data with the reference data into one file. Merging the reference dataset with the MODS dataset resulted in 8953 records, followed by merging with the NEMS dataset that resulted in 8924 records. There were a total of 157 (1.73 % of the original dataset) records missing. To obtain the ICD-10-CA data to calculate the modified CCS, the reference dataset was forwarded to the LHSC Records Department. A total of 8898 records were matched from Health Records, of which 183 records (2 % of the original dataset) from the CCIS dataset were not matched with the hospital records. This is likely due to error in the MRN number and/or failure to capture the ICU admission during discharge coding. Programmed SAS codes were created to extract only the type 1 diagnoses and calculation of the modified CCI score. Then, the dataset containing the modified CCI was combined with the final CCIS dataset (*N* = 8924) resulted in 8822 records for analysis, from which 2.9 % records were not captured from the original reference dataset, Fig. 1.

### Covariates associated with ICU mortality

Based on the literature review, the covariates associated with risk of mortality upon admission to the ICU included age, gender, MODS, NEMS, CCI, source of admission, ICU admission diagnosis, and ICU readmission during the same hospital admission. The continuous, nominal, and ordinal data of the covariates are categorized accordingly (Additional file 2: Table S2). The variable threshold used to divide it into the groups for analysis was done using the mean value for each of the variables for survivors and non-survivors as a reference point. Then, through much iteration with SAS 9.3, the developed groups for each of the variables that give the best discriminatory performances and Hosmer and Lemeshow goodness-of-fit were utilized.

### Statistical analysis

Univariate analysis was conducted for all baseline characteristics, and values were compared for survivors and non-survivors. For continuous variables, data are expressed as mean ± SD and comparisons conducted using the Student’s *t* test. For categorical variables, data are reported as proportions and comparison made using Pearson’s chi-square test. The prediction model for ICU mortality on admission was constructed by dividing the whole dataset into two random subgroups, “training” and “testing” set. To create the two random sample subgroups (training and testing set), the authors used the SAS 9.3 Ranuni function which generates a random number from a continuous uniform distribution with the interval (0,1) in which we used less than equal to 0.5 for group 1 and greater than 0.5 for group 2. Each 15th record will then be assigned to a random number. After assigning a random number to each record, it is then sorted in ascending or descending order of the random number assigned. A stepwise selection algorithm was also used to select from the eight covariates in the raw logistic regression prediction model. If the covariate significance was less than 0.05, it remained in the model; otherwise, the covariate exited the model. The remaining covariates were then fitted to a raw multivariable logistic regression model.

Often a predictive model’s estimate of these measures from the training set tends to overstate the predictive ability of the chosen model in another dataset. The amount of overestimation is referred to as the “optimism” of the estimate. To further obtain a valid criterion for the performance of the model, the logistic regression model then fit to the bootstrap sample and the corresponding value for the AUC was calculated. The fitted model was then applied to the original dataset, and the value of the AUC was recalculated. The differences in the values for the AUC provide an estimate of the optimism. This process is repeated 500 and 1000 times, and the results are averaged to provide a final bootstrap estimate for the optimism of the AUC.

The ability of the model to estimate mortality and agree with the actual outcome within groups of subjects of similar predicted risks by using the Hosmer and Lemeshow goodness-of-fit statistic obtained by grouping the subjects of the prediction model into *k* categories of percentiles. A good calibration is considered to be consistent with a small *χ*^2^ value for the Hosmer and Lemeshow test statistic.

All analyses were performed using SAS 9.3 (SAS Institute Inc., Cary, NC, USA). All tests presented are two-sided, and a *p* value <0.05 is considered significant.

## Results

Table 1 compares the baseline characteristics for survivors and non-survivors in a cohort of 8822 subjects. There were 5037 (57.10 %) males with mean age 60.67 ± 17.19 and 3785 (42.90 %) females with mean age 61.16 ± 17.66 with no mortality differences between males and females (23.23 % vs. 22.17 %; *p* = 0.24) but, the mean age was higher for non-survivors than survivors (66.82 vs. 59.13; *p* < 0.0001). The admission sources with the highest mortality included the wards, emergency department, and other hospital transfers whereas patients admitted to critical care post-operatively had the lowest mortality (*p* < 0.0001). There was also a statistically significant association between ICU mortality and ICU admitting diagnosis (*p* < 0.0001) with cardiovascular/cardiac/vascular diseases having the highest mortality and gastrointestinal disease, the lowest. The MODS and NEMS scores were both significantly associated with ICU mortality (*p* < 0.0001) whereas the modified CCI did not have a statistically significant association between survivors and non-survivors, *p* = 0.74. There was also no significant difference in mortality for those re-admitted back to the ICU during the same hospital admission compared to those that were not readmitted (9.19 % vs. 9.01 %; *p* = 0.81.).

**Table 1:** Baseline characteristics comparison between survivors and non-survivors of Critical Care Trauma Center (CCTC) and Medical-Surgical Intensive Care Unit (MSICU) at London Health Sciences Center (LHSC), between 1 Jan 2009 and 30 Nov 2012, *N* = 8822

Baseline characteristics	Survivors, *N* (%)	Non-survivors, *N* (%)	*p* value
Total number of subjects	6813 (77.23)	2009 (22.77)	
Sex			0.24
Male	3867 (56.76)	1170 (58.24)	
Female	2946 (43.24)	839 (41.76)	
Age, mean ± SD	59.13 ± 17.49	66.82 ± 15.67	<0.0001
Male ± SD	58.94 ± 17.29	66.40 ± 15.55	
Female ± SD	59.38 ± 17.76	67.40 ± 15.81	
0–39	1067 (15.66)	124 (6.17)	
40–79	4972 (72.98)	1452 (72.27)	
≥ 80	774 (11.36)	433 (21.55)	
Intensive care units			0.06
Critical Care Trauma Center (CCTC)	3542 (51.99)	1092 (54.36)	
Medical Surgical Intensive Care Unit (MSICU)	3271 (48.01)	917 (45.64)	
ICU admission source			<0.0001
Operating room/post-anesthesia care unit	1748 (25.66)	191 (9.51)	
Hospital—outside or within LHIN	1175 (17.25)	389 (19.36)	
Emergency department	1983 (29.11)	624 (31.06)	
Other source^a^	625 (9.18)	195 (9.71)	
Unit/ward	1280 (18.79)	610 (30.36)	
ICU admission diagnosis			<0.0001
Cardiovascular/cardiac/vascular	895 (13.14)	463 (23.05)	
Other diagnosis^b^	1727 (25.35)	353 (17.57)	
Gastrointestinal	826 (83.94)	158 (7.86)	
Respiratory	2091 (30.69)	655 (32.60)	
Trauma	473 (6.94)	106 (5.28)	
Neurological	801 (11.76)	274 (13.64)	
Multiple Organ Dysfunction Score (MODS)			<0.0001
Mean MODS ± SD	4.65 ± 2.96	7.01 ± 3.23	
Minimum-maximum MODS	0–19	0–20	
0	495 (7.27)	25 (1.24)	
1–4	3035 (44.55)	450 (22.40)	
5–8	2605 (38.24)	930 (46.29)	
9–12	591 (8.67)	492 (24.49)	
> 13	87 (1.28)	112 (5.57)	
Nine Equivalents Nursing Manpower Use Score (NEMS)			<0.0001
Mean NEMS ± SD	30.50 ± 8.45	36.64 ± 8.68	
Minimum-maximum NEMS	0–56	0–56	
0–22	990 (14.56)	94 (4.68)	
23–29	2458 (36.14)	388 (19.31)	
≥30	3353 (49.30)	1527 (76.01)	
Modified Charlson’s Comorbidity Index(CCI)			0.74
Mean CCI ± SD	1.30 ± 0.57	1.34 ± 0.61	
Score 0	5148 (75.56)	1461 (72.72)	
Score 1–3	1280 (18.79)	405 (20.16)	
Score >3	385 (5.65)	143 (7.12)	
Re-admission to ICU (same hospital admission)	626 (9.19)	181 (9.01)	0.81

^a^ Other Source includes patients admitted from the following locations: home—within or outside LHIN, level 2 unit or step-down unit, level 3 unit (medical/surgical or specialty unit), complex continuing care facility, rehabilitation facility, outside province, other

^b^Other diagnosis includes patients with the following diseases: metabolic/endocrine, genitourinary, musculoskeletal, skin, oncology, hematology, other

The baseline characteristics partitioned between the groups “training” and ‘testing’ sets were similar (Table 2). The total number of subjects in the training group was 4321 (48.98 %) as compared to 4501 (51.02 %) in the testing group. The combination of the patients from the two ICUs for each of the groups was evenly distributed between the two groups. In the training group, there were 2310 (53.46 %) subjects from CCTC and 2011 (46.54 %) from MSICU, while in the testing group, there were 2324 (51.63 %) from CCTC and 2177 (48.37 %) from MSICU.

**Table 2:** Training (*N* = 4321) and validation (*N* = 4501) dataset baseline characteristics for Critical Care Trauma Center (CCTC) and Medical-Surgical Intensive Care Unit (MSICU) at London Health Sciences Center (LHSC)

Baseline characteristics	Training, *N* (%)	Validation, *N* (%)
Total number of subjects	4321 (48.98)	4501 (51.02)
Sex		
Male	2475 (57.28)	2562 (56.92)
Female	1846 (42.72)	1939 (43.08)
Age		
0–39	582 (13.47)	609 (13.53)
40–79	3146 (72.81)	3278 (72.83)
≥80	593 (13.72)	614 (13.64)
ICU		
Critical Care Trauma Unit(CCTC)	2310 (53.46)	2324 (51.63)
Medical and Surgical Intensive Care Unit(MSICU)	2011 (46.54)	2177 (48.37)
ICU admission source		
Operating room/post-anesthesia care unit	932 (21.57)	1007 (22.38)
Hospital—outside or within LHIN	789 (18.26)	775 (17.23)
Emergency department	1264 (29.25)	1343 (29.85)
Other source^a^	379 (8.77)	441 (9.80)
Unit/ward	975 (22.15)	933 (20.74)
ICU admission diagnosis		
Cardiovascular/cardiac/vascular	652 (15.09)	706 (15.69)
Other diagnosis^b^	998 (23.10)	1082 (24.04)
Gastrointestinal	452 (10.46)	532 (11.82)
Respiratory	1405 (32.52)	1341 (29.79)
Trauma	287 (6.64)	292 (6.49)
Neurological	527 (12.20)	548 (12.18)
Multiple Organ Dysfunction Score (MODS)		
0	245 (5.67)	275 (6.11)
1–4	1725 (39.92)	1760 (39.10)
5–8	1733 (40.11)	1802 (40.04)
9–12	531 (12.29)	552 (12.26)
>13	87 (2.01)	112 (2.49)
Nine Equivalents Nursing Manpower Use Score (NEMS)		
0–22	557 (12.90)	527 (11.73)
23–29	1401 (32.45)	1445 (32.16)
≥30	2359 (54.64)	2521 (56.11)
Modified Charlson’s Comorbidity Score (CCI)		
0	3255 (75.33)	3354 (74.52)
1–3	813 (18.82)	872 (19.37)
>3	253 (5.86)	275 (6.11)
Re-admission to ICU (same hospital admission)	394 (9.12)	413 (9.18)
Mortality	986 (22.82)	1023 (22.73)

^a^ Other Source includes patients admitted from the following locations: home—within or outside LHIN, level 2 unit or step-down unit, level 3 unit (medical/surgical or specialty unit), complex continuing care facility, rehabilitation facility, outside province, other

^b^Other diagnosis includes patients with the following diseases: metabolic/endocrine, genitourinary, musculoskeletal, skin, oncology, hematology, other

In the multivariable logistic regression model, the stepwise selection algorithm eliminated the variables sex, *p* = 0.20 and readmit, *p* = 0.16. The *c*-statistic of the reduced model was smaller to that obtained when all explanatory variables were forced in (*c* = 0.774). The backward elimination algorithm eliminated readmit, *p* = 0.16 first, and then sex, *p* = 0.22, and resulted in a final model very similar to that using stepwise selection (*c* = 0.774). Because there is already a parsimony of variables in comparison to reported models, the two variables eliminated by both algorithms were forced back into the whole model.

The logistic regression analysis of the training group (Table 3) revealed that the model containing the explanatory variables compared to that with the intercept only significantly impacted the predictive ability of the model with the likelihood ratio, *χ*^2^ = 835.98, *p* < 0.0001. The overall effect of each of the covariates on mortality revealed that all except gender (*χ*^2^ = 0.59; *p* = 0.44) and CCI (*χ*^2^ = 4.60; *p* = 0.10) had a significant independent effect on ICU mortality. Categorically, the odds of mortality are much higher for older patients, specifically ages 40–79 that is 2.23 (95 % confidence interval (CI) 1.64, 3.04), and those of patients ≥80 years old was 5.51 (95 % CI 3.87, 7.84) times higher than those of patients age ≤ 39. For patients admitted to the ICUs from the unit/ward (odds ratio (OR) = 4.93; 95 % CI 3.69, 6.59), other hospitals (OR = 3.054; 95 % CI 2.26, 4.12), emergency departments (OR = 2.71; 95 % CI 2.05, 3.59), and specialty units (OR = 2.66; 95 % CI 1.86, 3.81) had higher odds of ICU mortality as compared to patients admitted from the operating room/post-anesthesia unit. Patients admitted with cardiovascular/cardiac/vascular diagnoses had a higher mortality as compared to other etiologies. Higher scores in MODS and NEMS corresponded to increasing ICU mortality (*p* ≤ 0.0001). There was a weak association with ICU readmission and mortality (OR = 0.742; 95 % CI 0.56, 0.99; *p* = 0.04).

**Table 3:** Multivariable logistic regression analysis of the training set (*N* = 4321) for Critical Care Trauma Center (CCTC) and Medical-Surgical Intensive Care Unit (MSICU) at London Health Sciences Center (LHSC)

	Beta (β) coefficient	Wald chi-square	Odds ratio (95 % CI)	*p* value
Intercept	−5.18	137.10	–	<.0001
Age				<.0001
0–39 (reference)	–	–	–	–
40–79	0.80	25.73	2.23 (1.64–3.04)	
≥80	1.71	90.17	5.51 (3.87–7.84)	
Sex				0.44
Male (reference)	–	–	–	–
Female	0.06	0.60	1.07 (0.91–1.25)	
Nine Equivalents Nursing Manpower Use Score (NEMS)				<.0001
0–22 (reference)	–	–	–	–
23–29	0.39	4.79	1.48 (1.04–2.10)	
≥30	1.02	36.57	2.77 (1.99–3.84)	
Multiple Organ Dysfunction Score (MODS)				<.0001
0 (reference)	–	–		–
1–4	1.18	9.87	3.25 (1.56–6.78)	
5–8	1.91	26.24	6.78 (3.26–14.09)	
9–12	2.90	57.39	18.09 (8.55–38.25)	
≥13	3.56	65.11	35.16 (14.81–83.46)	
ICU Admission Source				<.0001
Operating room/post-anesthesia care unit (reference)	–	–	–	
Other source^a^	0.98	28.56	2.66 (1.86–3.81)	
Emergency department	1.00	48.76	2.71 (2.05–3.59)	
Hospital—outside or within LHIN	1.12	58.76	3.05 (2.26–4.12)	
Unit/ward	1.60	115.846	4.93 (3.69–6.59)	
ICU admission diagnosis				<.0001
Cardiovascular/cardiac/vascular (reference)	–	–	–	–
Other diagnosis^b^	−0.81	37.67	0.45 (0.34–0.58)	
Gastrointestinal	−0.80	20.16	0.45 (0.32–0.64)	
Respiratory	−0.56	22.85	0.57 (0.45–0.72)	
Trauma	−0.32	2.52	0.73 (0.49–1.08)	0.11
Neurological	0.002	0.0002	1.00 (0.76–1.33)	0.99
Re-admission to ICU (same hospital admission)	−0.30	4.02	0.74 (0.56–0.99)	0.04
Modified Charlson’s Comorbidity Index (CCI)				0.1
0 (reference)	–	–	–	–
1–3	−0.21	4.20	0.81 (0.66–0.99)	
>3	0.05	0.10	1.06 (0.76–1.48)	

^a^Other Source includes patients admitted from the following locations:home—within or outside LHIN, level 2 unit or step-down unit, level 3 unit (medical/surgical or specialty unit), complex continuing care facility, rehabilitation facility, outside province, other

^b^Other diagnosis includes patients with the following diseases: metabolic/endocrine, genitourinary, musculoskeletal, skin, oncology, hematology, other

**Fig. 2:**
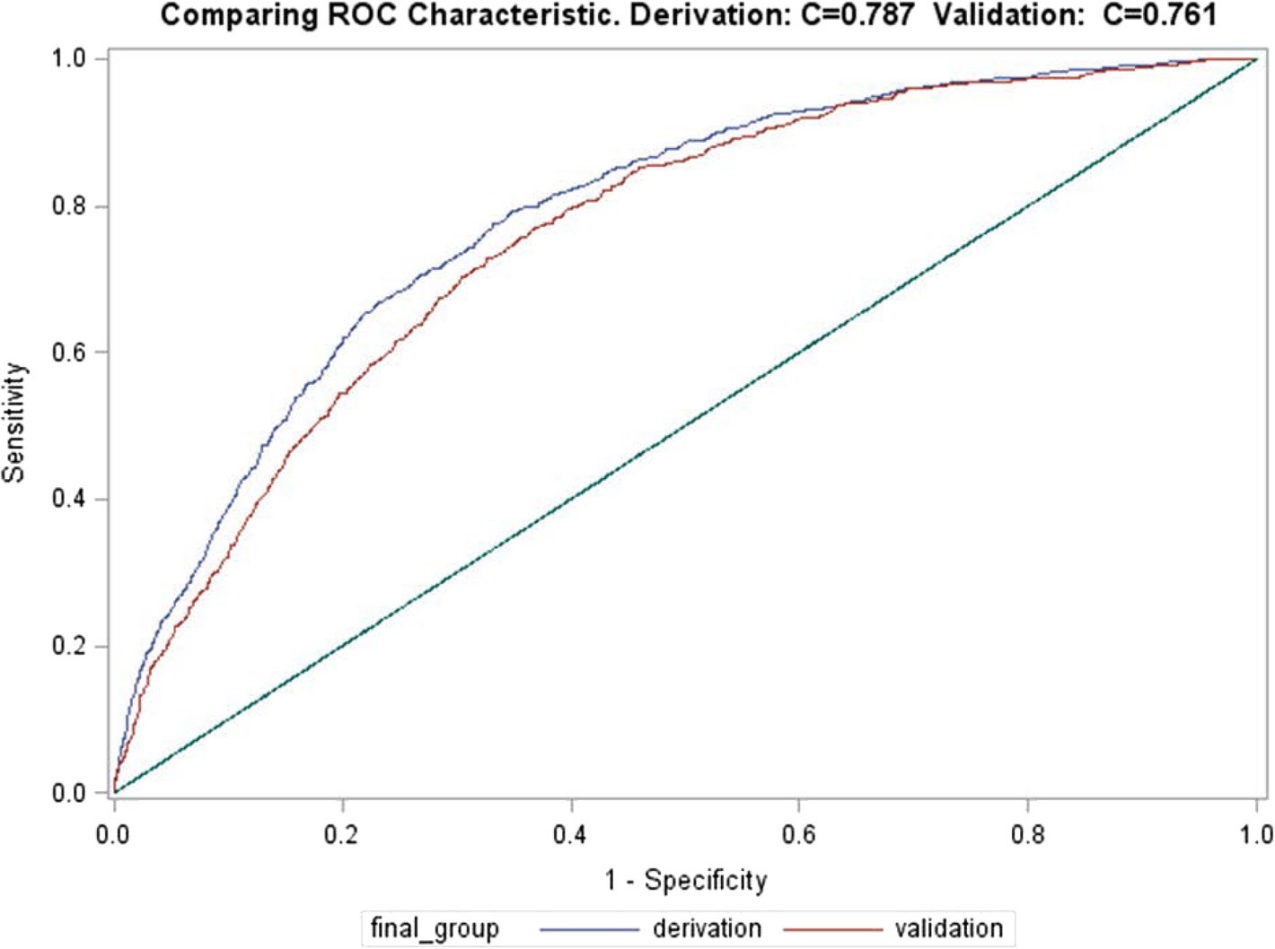
Comparison of the receiver operating curve (ROC) for the training dataset (*red*) and the testing dataset (*blue*). The area under the curve (AUC) was 0.787 for the training dataset and 0.760 for the validation dataset

The discriminatory performance of the training model revealed the AUC was 0.787. This indicates that the model has good ability to distinguish between patients with a high risk of mortality and those with a low risk of mortality [15]. The comparison of the receiver operating curve (ROC) curves for the training dataset and the testing dataset indicated an area difference of 0.026 (0.787–0.761), which reflects a very narrow gap or the optimism between the two curves, suggesting a small degradation in the model’s performance in prospective testing (Fig. 2). To validate this difference, the bootstrap processes were repeated 500 and 1000 times, and the results were averaged to provide an optimism correction for the AUC of 0.003 (AUC range = 0.758–0.790) which indicates that our model does not overpredict (Additional file 3: Table S3). The AUC comparison between this new model with MODS and NEMS alone in the new model revealed AUC = 0.776 and 0.736, respectively, which are lower than the combined scores AUC = 0.787 (Additional file 4: Table S4). Overall, the combination of the two scores in the model gives better discrimination ability between patients with high and low risks for ICU mortality during the first 24 h of ICU admission.

As a measure of calibration from the model, the Hosmer and Lemeshow goodness-of-fit statistics revealed *χ*^2^ = 5.48 (*p* > 0.31) indicating strong agreement between observed and expected ICU mortality (Table 4).

**Table 4:** Hosmer and Lemeshow and goodness-of-fit test for the multivariable logistic regression model

Partition for the Hosmer and Lemeshow test						
			Mortality = 1		Mortality = 0	
Group	Range of predicted mortality probability	Total	Observed	Expected	Observed	Expected
1	0–3.58 %	433	11	9.73	422	423.27
2	3.59–6.26 %	432	21	22.03	411	409.97
3	6.27–9.56 %	432	31	33.91	401	398.09
4	9.57–13.06 %	434	45	49.05	389	384.95
5	13.07–18.24 %	432	66	66.83	366	365.17
6	18.25–23.35 %	432	91	90.61	341	341.49
7	23.36–28.25 %	432	108	109.35	324	320.84
8	28.26–39.11 %	438	174	149.02	264	288.98
9	39.12–49.38 %	435	190	190.70	245	244.30
10	49.39–94.08 %	417	249	262.96	168	154.04
Hosmer and Lemeshow goodness-of-fit test						
Chi-square 9.3599			*p* value 0.3128			

The final formula equation for our model is

Log [Mortality (at 24 h ICU admission)] = −5.18 + 0.80[age(40–79)] + 1.71[age(>80)] + 0.60[Sex(male = 0 and female = 1) + 0.98[Other source admission] + 0.00[Operating room/post-anesthesia care] + 1.00[ER admission] + 1.12[Hospital-outside or within LHIN] + 1.60[Ward admission]Cardiovascular/Cardiac/Vascular] + 0.00[−0.81[Other diagnosis] − 0.80[Gastrointestinal] − 0.56[Respiratory] − 0.32[Trauma] + 0.002[Neurological] − 0.30[ICU re-admission] − 0.21[CCI(1–3)] + 0.05[CCI(>3)] + 0.0[NEMS(0–22)] + 0.39[NEMS(23–29)] + 1.02[NEMS(≥300] + 1.18[MODS(1–4)] + 1.91[MODS(5–8)] + 2.90[MODS(9–120] + 3.56[MODS(≥130].

## Discussion

Currently, many health care performance measurement systems are based on administrative databases. These systems are often developed to fulfill the needs of funding agencies and support the quality improvement plans of individual hospitals but rarely provide the necessary level of risk adjustment to provide meaningful comparison, over time or across facilities. It is also well known that prognostic research has received limited attention as compared to etiological, diagnostic, and therapeutic research. The development and application of robust prognostic models are essential for valid benchmarking. Prognostic scoring systems have been developed by the critical care specialty in an effort to quantify the severity of illness of a given patient or groups of patient [16–18]. Adjustment for the severity of illness enables one to monitor the performance of an ICU over time and to allow comparison of ICUs in the same or different hospitals. However, the fact that many prognostic models currently exist suggests that the optimum model has not yet been fully established and any of the developed prognostic models will have a limited effective life span [16, 19], due to changes in clinical practice over time and improved health care that can alter the risk of mortality for a given clinical situation. Thus, prognostic models require periodic updating. Major revisions of prognostic models that were published between 2005 and 2007 include APACHE IV (AUC = 0.88, *χ*^2^ = 16.9, *p* = 0.08) [20], SAPS 3 (AUC = 0.848, *χ*^2^ = 14.29, *p* = 0.16) [21], and MPM_0_ III (AUC = 0.823, *χ*^2^ = 11.62, *p* = 0.31) [22]. A recent review evaluated the latest versions of these models and concluded that although they represent great improvement compared to the previous ones, regular updates and local customizations are required [23]. Also, the huge resources burden needed to collect a significantly large amount of data for the variables to generate these scores is daunting. The present study aimed to use available data already collected by our ICUs and other ICUs for a very limited number of variables for the two scores, MODS and NEMS in the province of Ontario, Canada, as mandated by CCIS as well as comorbidities diagnoses collected by our hospital health records to develop a mortality prognostic model. The AUC for our model was 0.787, which is considered acceptable or very good in differentiating between survivors and non-survivors [24, 25]. This model is well calibrated, showing good agreement between predicted and actual outcomes for all risk strata (Hosmer and Lemeshow *χ*^2^ = 5.4761, *p* = 0.3146) [26].

To the best of our knowledge, this study is the first to use validated organ dysfunction score, MODS and severity assessment based on nursing workload, and NEMS coupled with five other variables selected. Overall, only two out the seven independent variables, gender and CCI, were not significant in the prediction of ICU mortality. Two retrospective studies contradicted our findings with respect to gender. One study of 24,778 patients admitted to the ICUs across Ontario, Canada in 2001–2002 revealed that females had a higher ICU mortality than males with an adjusted OR = 1.20 (95 % CI 1.10–1.31, *p* < 0.001) [27]. Another study of 18,757 patients diagnosed with sepsis in 98 ICUs between 2003 and 2006 reported an adjusted OR = 1.11 (95 % CI 1.04–1.19, *p* < 0.01) [28]. This increased mortality in female patients was not fully understood but could be explained by differences in the presentation of critical illness, decision-making, or unmeasured confounding factors that may contribute to these findings. The other possibility that our patient cohort was reported at a later time period may result in improved ICU access and earlier care of patients using the Critical Care Outreach Team (CCRT) [29–31].

We know that scoring systems used in the ICUs have been introduced and developed over the last 30 years. These models allow an assessment of the severity of disease and provide an estimate of ICU and hospital mortality. The MODS score independently has been used in many clinical studies and it has an excellent discriminating predictor of mortality in ICU patients [32–37]. The MODS in our study is a very strong predictor of mortality in the first 24 h of ICU admission, and it correlated very well with other scores such as the SOFA score and APACHE II score in terms of mortality prediction [38]. However, the complexity of ICU care goes beyond the severity of illness or organ failure, the level of nursing workload, and NEMS as related to the ICU resource utilization that also correlated well with ICU mortality [39]. Many other factors also have been shown to increase risks of in-hospital mortality after admission to the ICU, including increasing age and severity of acute illness, certain pre-existing medical conditions, source of admission, physiological measurements, and biochemical/hematological indices [40]. By utilizing those other covariates, it may not necessarily improve the discrimination ability of predicted model but rather avoid the pitfall of either underpredicting or overpredicting ICU mortality when using only a single covariate in the prediction model. Although the NEMS in our study is not as a strong mortality predictor in the first 24 h of ICU admission as the MODS, with the combined scores in a prediction model, it provided a much better basis for evaluation of treatment results and documentation of the ICUs’ resource needs [39]. Having knowledge of both severity of organ dysfunction and degree of resource utilization will provide a better basis for assessing whether ICU treatment(s) and/or administrative protocol(s) needs to be modified to improve patient care.

With comorbidities, specifically severe chronic organ system insufficiency or immunocompromised, those patients markedly influence outcomes [41] and this is supported by other outcome prediction scores. In our study, comorbidities were not predictive because we used the pre-admit comorbidities that existed prior to admission to the ICU as opposed to the conditions that were aggravated or developed subsequently. Another limitation was the actual condition captured in the CCI score that was developed on breast cancer patients and not in ICU patients to predict 1-year patient mortality using comorbidity data obtained from hospital chart review [42, 43]. The CCI is a validated weighted score, the weight for each of the co-morbidities may not fully reflect the severity of the disease and it may or may not include specific or unusual illnesses, and therefore, it is not an assessment of the impact of all illnesses on the overall health of the patient. Furthermore, the ICD-10-CA data is abstracted by medical record clerks and not entered by health care providers and can be subjected to errors based on lack of documentation and misinterpretation. Our model fit could be improved by capturing active chronic health status at ICU admission utilizing the APACHE II chronic health points which reflects diminished physiological reserve and markedly influence outcome [2, 41].

Acute diagnosis was not used in earlier prediction models with the exception of the APACHE II to IV scores. It was not until 1993 that MPM II started to include acute diagnosis to the model and SAPS 3 and MPM III followed suit in 2005 and 2007. However, the predictive accuracy over diagnosis showed that the performance of a prediction model can vary in different diagnostic groups [44]. This is in agreement with previous research [45–47], which suggested that prognostic models can underpredict or overpredict mortality in specific patient sub-groups. The admitting diagnosis in our study is classified using broad, system-based categories and did not include specific diagnostic information to allow for comparisons within the generalized diagnoses, between subgroups or between-study populations. Although these broad diagnostic groups include specific diagnoses that are similar based on the system involved, the exact diagnoses within a group can have completely different treatments and outcomes. For example, the “cardiovascular/cardiac/vascular” diagnosis group used in this study has the highest mortality but includes less severe exact diagnoses with lower mortality rates. Patients with abdominal aorta aneurysm carried a much higher mortality than patients with myocardial infarction and cardiac bypass surgery thus skewing the mortality risk. Another limitation, some of the system-based groups such as metabolic/endocrine, genitourinary, musculoskeletal, skin, oncology, hematology, and “other” was collapsed together due to its small number of patients per group. This represented a significant heterogeneous population within a diagnostic group which would be difficult to interpret the statistical prediction accuracy.

Studies carried out in numerous countries indicated that the source of the patient admission is associated with mortality. Patients transferred from the ward within the same hospital showed a greater ICU mortality when compared with those coming from other sources [48–51]. This is in agreement with the present study where patients admitted from the ward had the highest mortality (OR = 4.93, 95 % CI 3.69–6.59, *p* < 0.0001), post-surgical patients had the lowest.

Those patient readmitted to the ICU did not have significant mortality differences to those patients not readmitted, which is contrary to published literature [52]. This difference could be due to the implementation of the Critical Care Resuscitation Team (CCRT) that may intervene earlier on the wards of patients’ acute illness [53, 54]. Also, various service teams including CCRT are improving end of life (EOL) discussions with those patients previously admitted to the ICU, thus avoids a readmission [55, 56]. Our institution implemented the CCRT service in 2007.

## Conclusions

Scoring systems in critical care have evolved to meet the desire of clinical and administrative leaders to assess the quality of care provided by the ICUs. Mortality is a key ICU quality metric and reflects many aspects of ICU care, including use of best practice, accurate diagnosis, and effective and timely therapies. Our model is locally calibrated to two ICUs in London, Ontario, Canada only, and the results may not be generalizable to other critical care units. But collectively, all ICUs in the province of Ontario, Canada gather the same data information; it is logical that a model be developed to benchmark ICU performance and improve the usability of the current reporting system. This study demonstrates that data from the CCIS can be used to create a mortality prediction model with good calibration and discrimination. Inclusion of data to capture active chronic health status and refinement of the acute diagnosis classification could further improve predictive ability of the developed model.

## Additional files

Additional file 1: Table S1. Nine equivalents of nursing manpower use score by Miranda DR, Nap R, de RA, Schaufeli W, Iapichino G. Nursing activities score. Crit Care Med 2003;31:374–382. (DOCX 87 kb) Additional file 2: Table S2. Selection and categorization of the independent variables for the logistic regression model associated with ICU mortality. (DOCX 14 kb) Additional file 3: Table S3. Bootstrap sampling (500 and 1000 times) to estimate the optimism in the AUC (*c*-statistics) based on the predicted outcomes from a logistic regression model. (DOCX 13 kb) Additional file 4: Table S4. Multivariable logistic regression analysis of the training set (*N* = 4321) for Critical Care Trauma Center (CCTC) and Medical-Surgical Intensive Care Unit (MSICU) at London Health Sciences Center (LHSC) with MODS or NEMS only. (DOCX 14 kb)

## Abbreviations

APACHE: Acute physiology and chronic health evaluation
AUC: Area under the curve
CCI: Charlson’s Co-Morbidity Index
CCIS: Critical care information system
CCSO: Critical care services Ontario
CCTC: Critical care trauma centre
CI: Confidence interval
CIHI: Canadian Institute of Health Information
ICD-10-CA: International statistical classification of diseases and related health problems – tenth version – Canada
ICU: Intensive care unit
LHIN: Local health information networks
LHSC: London health sciences centre
MODS: Multiple organs dysfunction score
MPM: Mortality prediction model
MSICU: Medical and surgical intensive care unit
NEMS: Nine equivalent manpower use score
OR: Odds ratio
ROC: Receiver operating curve
SAPS: Simplified acute physiological score
SOFA: Sequential organ failure assessment
TISS: Therapeutic intervention scoring system
