# Correction to: To develop a regional ICU mortality prediction model during the first 24 h of ICU admission utilizing MODS and NEMS with six other independent variables from the critical care information system (CCIS) Ontario, Canada

**DOI:** 10.1186/s40560-019-0421-1

**Published:** 2020-01-14

**Authors:** Raymond Kao, Fran Priestap, Allan Donner

**Affiliations:** 1 Department of National Defense, Royal Canadian Medical Services, 1745 Alta Vista Drive, Ottawa, Ontario K1A 0K6 Canada; 2 London Health Sciences Center, Divisions of Critical Care and Robarts Research Institute, Western University, 800 Commissioner’s Rd E, London, Ontario N6A 5W9 Canada; 3 Harvard School of Public Health, Harvard University, 677 Huntington Ave, Boston, MA 02115 USA

**Correction to: J Intensive Care**

**https://doi.org/10.1186/s40560-016-0143-6**

In the original publication of this article [1], there were several transcription errors in the logistic regression equation model as below:

- **Equation published:**
  Log [Mortality (at 24 h ICU admission)] = −5.18 + 0.80[age(40–79)] + 1.71[age(>80)] + 0.60[Sex(male = 0 and female = 1) + 0.98[Other source admission] + 0.00[Operating room/post-anesthesia care] + 1.00[ER admission] + 1.12[Hospital-outside or within LHIN] + 1.60[Ward admission] Cardiovascular/Cardiac/Vascular] + 0.00[−0.81[Other diagnosis]− 0.80[Gastrointestinal] − 0.56[Respiratory] − 0.32[Trauma] + 0.002[Neurological] − 0.30[ICU re-admission] − 0.21[CCI(1–3)] + 0.05[CCI(>3)] + 0.0[NEMS(0–22)] + 0.39 [NEMS(23–29)] + 1.02[NEMS(≥300] + 1.18[MODS(1–4)] +1.91[MODS(5–8)] + 2.90[MODS(9–120] + 3.56[MODS(≥130].

b. **Corrected equation:**
  $$ Logit\ \left[ Mortality\ \left( at\ 24\ h\  ICU\  admission\right)\right]=-5.41+0.00\left[ age\left(0-39\right)\right]+0.80\left[ age\ \left(40-79\right)\right]+1.71\left[ age\left(>80\right)\right]-0.06\left[ Sex\ \left( male=0\  and\ female=1\right)\right]+0.98\left[ Other\ source\ admission\right]+0.00\left[ Operating\ room/ post\ anesthesia\ care\right]+1.00\left[ ER\  admission\right]+1.12\ \left[ Hospital- outside\ or\ within\ LHIN\right]+1.60\left[ Ward\ admission\right]+0.00\left[ Cardiovascular/ Cardiac/ Vascular\right]-0.81\left[ Other\ diagnosis\right]-0.80\left[ Gastrointestinal\right]-0.56\left[ Respiratory\right]-0.32\ \left[ Trauma\right]+0.002\left[ Neurological\right]+0.30\left[ ICU\  re- admission\right]-0.21\left[ CCI\ \left(1-3\right)\right]+0.05\left[ CCI\left(>3\right)\right]+0.0\left[ NEMS\ \left(0-22\right)\right]+0.39\ \left[ NEMS\ \left(23-29\right)\right]+1.02\left[ NEMS\left(\ge 30\right)\right]+1.18\left[ MODS\ \left(1-4\right)\right]+1.91\left[ MODS\ \left(5-8\right)\right]+2.90\left[ MODS\ \left(9-12\right)\right]+3.56\left[ MODS\ \left(\ge 13\right)\right]. $$

The authors sincerely apologize for the inconvenience caused to the readers.
