# Supplementary material for: To develop a regional ICU mortality prediction model during the first 24 h of ICU admission utilizing MODS and NEMS with six other independent variables from the Critical Care Information System (CCIS) Ontario, Canada
Source: J Intensive Care. 2016 Feb 29;4:16. doi: 10.1186/s40560-016-0143-6 (PMC4772333; doi:10.1186/s40560-016-0143-6)
Supplement: Additional file 1: Table S1. — Nine equivalents of nursing manpower use score by Miranda DR, Nap R, de RA, Schaufeli W, Iapichino G. Nursing activities score. Crit Care Med 2003;31:374–382. (DOCX 87 kb) [file 40560_2016_143_MOESM1_ESM.docx]

**Additional file 1: Table S1**. Nine Equivalents of Nursing Manpower use Score by Miranda DR, Nap R, de RA, Schaufeli W, Iapichino G. Nursing activities score. *Crit Care Med* 2003;31:374-382
