# Supplementary material for: To develop a regional ICU mortality prediction model during the first 24 h of ICU admission utilizing MODS and NEMS with six other independent variables from the Critical Care Information System (CCIS) Ontario, Canada
Source: J Intensive Care. 2016 Feb 29;4:16. doi: 10.1186/s40560-016-0143-6 (PMC4772333; doi:10.1186/s40560-016-0143-6)
Supplement: Additional file 2: Table S2. — Selection and categorization of the independent variables for the logistic regression model associated with ICU mortality. (DOCX 14 kb) [file 40560_2016_143_MOESM2_ESM.docx]

| **Independent variables associated with ICU mortality** | **Categorize the independent variable** |
| --- | --- |
| Age (years) | 18-39 (Reference group) |
|  | 40-80 |
|  | ≥ 80 |
| Gender | Male = 0 (Reference group) |
|  | Female=1 |
| Multiple Organ Dysfunction Score (MODS) | 0 (Reference group) |
|  | 1-4 |
|  | 5-8 |
|  | 9-12 |
|  | ≥13 |
| Nine Equivalents of Nursing Manpower Use Score (NEMS) | 0-22 (Reference group) |
|  | 23-29 |
|  | ≥ 30 |
| Modified Charlson Co-morbidity Index (CCI) | 0 (reference group) |
|  | 1 |
|  | ≥ 3 |
| Source of admission to the ICU | Operating room(OR)/Post Anesthesia Unit (PACU) (Reference group) |
|  | Hospital – Outside & within Local Health Integration Network (LHIN) |
|  | Emergency department |
|  | Unit/Ward |
|  | Other: Home- outside and within LHIN, Level 2 Unit or Step Down Unit, Level 3 Unit (Medical/Surgical or Specialty Unit), Complex Continuing Care Facility within LHIN, Rehab Facility-Within LHIN, Inpatient–Rehab, Outside province |
| Admission diagnosis | Cardiovascular/Cardiac/Vascular (Reference group) |
|  | Gastrointestinal |
|  | Respiratory |
|  | Trauma |
|  | Neurological |
|  | Other: Metabolic / Endocrine, Genitourinary, Musculoskeletal / Skin, Oncology / Hematology |
| ICU readmission during the same hospital admission | No=0 (Reference group) |
|  | Yes=1 |

**Additional file 2: Table S2**. Selection and categorization of the independent variables for the logistic regression model associated with ICU mortality.
