# Supplementary material for: To develop a regional ICU mortality prediction model during the first 24 h of ICU admission utilizing MODS and NEMS with six other independent variables from the Critical Care Information System (CCIS) Ontario, Canada
Source: J Intensive Care. 2016 Feb 29;4:16. doi: 10.1186/s40560-016-0143-6 (PMC4772333; doi:10.1186/s40560-016-0143-6)
Supplement: Additional file 3: Table S3. — Bootstrap sampling (500 and 1000 times) to estimate the optimism in the AUC (c-statistics) based on the predicted outcomes from a logistic regression model. (DOCX 13 kb) [file 40560_2016_143_MOESM3_ESM.docx]

**Additional file 3: Table S3.** Bootstrap sampling (500 and 1000 times) to estimate the optimism in the AUC (c-statistics) based on the predicted outcomes from a logistic regression model.

| c-statistic | Bootstrap sampling | Mean AUC | Std Dev | Minimum AUC | Maximum AUC |
| --- | --- | --- | --- | --- | --- |
| C | 500 | 0.775 | 0.006 | 0.785 | 0.790 |
| C_Diff_ | 500 | 0.003 | 0.006 | -0.014 | 0.019 |
| C | 1000 | 0.775 | 0.006 | 0.757 | 0.791 |
| C_Diff_ | 1000 | 0.003 | 0.006 | -0.015 | 0.019 |
