# Supplementary material for: To develop a regional ICU mortality prediction model during the first 24 h of ICU admission utilizing MODS and NEMS with six other independent variables from the Critical Care Information System (CCIS) Ontario, Canada
Source: J Intensive Care. 2016 Feb 29;4:16. doi: 10.1186/s40560-016-0143-6 (PMC4772333; doi:10.1186/s40560-016-0143-6)
Supplement: Additional file 4: Table S4. — Multivariable logistic regression analysis of the training set (N = 4321) for Critical Care Trauma Center (CCTC) and Medical-Surgical Intensive Care Unit (MSICU) at London Health Sciences Center (LHSC) with MODS or NEMS only. (DOCX 14 kb) [file 40560_2016_143_MOESM4_ESM.docx]

**Additional file 4: Table S4**. Multivariable logistic regression analysis of the training set (N=4321) for Critical Care Trauma Center (CCTC) and Medical-Surgical Intensive Care Unit (MSICU) at London Health Sciences Center (LHSC) with MODS or NEMS only.

| **Model with MODS only** | **Beta (β) Coefficient** | **Wald Chi-Square** | **Odds Ratio (95% CI)** | **P-Value** |
| --- | --- | --- | --- | --- |
| **Nine Equivalents Nursing Manpower Use Score (NEMS)** |  |  |  |  |
| 0-22 **(reference)** | --------- | --------- | --------- | --------- |
| 23-29 | --------- | --------- | --------- | --------- |
| ≥ 30 | --------- | --------- | --------- | --------- |
| **Multiple Organ Dysfunction Score (MODS)** |  |  |  | <.0001 |
| 0 **(reference)** | --------- | ---------- | --------- | -------- |
| 1-4 | 1.18 | 14.38 | 4.10(1.98-8.51) |  |
| 5-8 | 1.91 | 37.64 | 9.64(4.68-19.88) |  |
| 9-12 | 2.90 | 78.01 | 27.91(13.33-58.41) |  |
| ≥13 | 3.56 | 88.23 | 58.52(25.03-136.80) |  |
| **Model with NEMS only** |  |  |  |  |
| **Nine Equivalents Nursing Manpower Use Score (NEMS)** |  |  |  | <.0001 |
| 0-22 **(reference)** | -------- | --------- | -------- | -------- |
| 23-29 | 0.39 | 11.67 | 1.80(1.28-2.53) |  |
| ≥ 30 | 1.02 | 89.53 | 4.56(3.33-6.24) |  |
| **Multiple Organ Dysfunction Score (MODS)** |  |  |  | <.0001 |
| 0 **(reference)** | --------- | --------- | --------- | -------- |
| 1-4 | --------- | --------- | --------- |  |
| 5-8 | --------- | --------- | --------- |  |
| 9-12 | --------- | --------- | --------- |  |
| ≥13 | --------- | --------- | --------- |  |
